# Prevention and Early Detection of Head and Neck Squamous Cell Cancers

**DOI:** 10.1155/2011/318145

**Published:** 2012-08-23

**Authors:** Toru Nagao, Pankaj Chaturvedi, Ashok Shaha, Rengaswamy Sankaranarayanan

**Affiliations:** ^1^Department of Oral and Maxillofacial, Okazaki City Hospital, Okazaki, Japan; ^2^Head and Neck Service, Tata Memorial Hospital, Mumbai, India; ^3^Head and Neck Service, Memorial Sloan-Kettering Cancer Center, New York, NY 10065, USA; ^4^Early Detection and Prevention Section (EDP), International Agency for Research on Cancer, 69372 Lyon, France

Head and neck squamous cell cancer (HNSCC) is the sixth most common malignancy reported worldwide and one with high mortality ratios among all malignancies. Unfortunately 5-year survival rate has not improved (50% overall) for the last few decades except in specialized cancer centres. Oral and HNSCC patients come at late stage due to their own delay as well as professional delay at primary and specialized care levels. Efficacious preventive strategies, educations, and early detections may have the capacity for HNSCC and potentially malignant disorders to be detected at an asymptomatic phase. For primary and secondary cancer prevention, changing lifestyle is an integral part of health promotion interventions, particularly among high risk group. Chemoprevention is an ideal one to lower the chance of getting cancer. Thus the early detection and subsequent intervention may achieve a significant reduction of mortality rate in this population.

 This special issue about prevention and early detection of HNSCC is all in our minds, and it stresses on current topics of epidemiology, diagnosis, tumour markers, and chemoprevention for oral and HNSCC. All eight papers are review articles from various parts of the world where oral and HNSCCs are major public health issues, although the incidence is not high.

With regards to tumour markers, no significant serological markers are available so far that would be helpful in detecting primary HNSCCs at early stage, but the most widely accepted biomarker for HNSCCs is high-risk HPV status. The incidence of oropharyngeal SCC is rising, and the Agency for Research on Cancer (IARC) now recognizes HPV as a risk factor for oropharyngeal cancer. L. Marklund and L. Hammarstedt present a current advancement of HPV studies in HNSCC: HPV biology, oncogenic mechanisms, risk factors, epidemiology, and clinical implications. HPV-positive oropharyngeal cancer is recognized as a distinct subset of HNSCC with a favourable outcome, and patients with HPV-positive oropharyngeal cancers often are younger and in good health. The authors suggest that further knowledge about tumour biology and the identification of additional clinical useful markers is needed to combine with HPV status for appropriate risk stratification in future clinical trials in order to optimize the treatment for each individual patient. X. Li et al. present an inhibitor of growth gene (*ING*) family consisting of five genes, from *ING1* to *ING5*, identified as a new tumour suppressor gene family. These* ING* family genes are supposed to belong to type II tumour suppressor gene and are involved in multiple cellular processes including chromatin remodeling, DNA repair, cell cycle control, senescence, and apoptosis. The authors conclude that the *ING* gene family could be a novel p53-independent biomarker for HNSCC.

 T. Tanaka and R. Ishigamori provide a review of the detection of high risk patients by potential biomarkers for oral carcinogenesis such as epidermal growth factor receptor (EGFR), which plays critical roles in HNSCC carcinogenesis and others well-known ones as well as chemoprevention. Individualized medical therapy to specific genetic abnormalities detected within the oral mucosa is a promising approach. M. Masuda et al. present a potential of green tea extract, (-)-epigallocatechin-3-galate (EGCG), in HNSCC chemoprevention and the role of EGFR signaling and lipid raft. The authors show the inhibition of EGFR by EGCG and the important role of lipid raft that emerged as an important platform of numerous biophysical functions such as receptor tyrosine kinase signaling including EGFR. Y. Zhao et al. present a Salvianolic acid B (Sal-B), which is a well-known Chinese medicine used to treat and prevent aging diseases for thousands of years and significantly inhibits or delays the growth of HNSCC in both cultured HNSCC cells and HNSCC xenograft animal models. Anticancer mechanisms such as inhibition of COX-2/PGE-2 pathway, promotion of apoptosis, and modulation of angiogenesis are proposed, and it is concluded that Sal-B is a potential HNSCC chemopreventive agent working through antioxidation and anti-inflammation mechanisms.

The adjunct diagnostic technique is important for early detection mostly at primary care level prior to tissue biopsy. S. F. Mendes et al. present a review of diagnostic techniques for oral potentially malignant disorders and oral exfoliative cytology, cytomorphometry, tissue staining, chemiluminescence, and light emission technique. A. Böcking et al. provide a useful method of brush biopsy and DNA image cytometry as screening tools for prevention, diagnosis, therapy, and follow-up care of oral cancer and precursor lesions. The authors suggest that the diagnostic DNA image cytometry is an accurate method and has internationally been standardized and noted that it is paid by the German health insurances. C. Szeto et al. try to review the efficacy of contact endoscopy by literature searches in early diagnosis, monitoring, and preoperative assessment of mucosal lesions of HNSCC. 

Oral and HNSCC is one of the lethal diseases in all cancers, and their natural history is still further behind. We hope that this issue may inspire the development of new strategies and policies for early detection and subsequent intervention for HNSCC in order to get better outcome of cancer prevention and treatment and, consequently, reduce the mortality rate.

*Toru Nagao* *Toru Nagao* *Pankaj Chaturvedi* *Pankaj Chaturvedi* *Ashok Shaha* *Ashok Shaha* *Rengaswamy Sankaranarayanan* *Rengaswamy Sankaranarayanan*

